# Boron nitride nanotubes: synthesis and applications

**DOI:** 10.1186/s40580-018-0149-y

**Published:** 2018-06-28

**Authors:** Jun Hee Kim, Thang Viet Pham, Jae Hun Hwang, Cheol Sang Kim, Myung Jong Kim

**Affiliations:** 1 Applied Quantum Composites Research Center, Korea Institute of Science and Technology, Wanju, 55324 Republic of Korea; 2 Department of Bionanosystem Engineering, Graduate School, Chonbuk National University, Jeonju, 54896 Republic of Korea; 3 Division of Mechanical Design Engineering, Chonbuk National University, Jeonju, 54896 Republic of Korea

**Keywords:** Boron nitride nanotubes, Synthesis methods, Scalable synthesis, Applications

## Abstract

Boron nitride nanotube (BNNT) has similar tubular nanostructure as carbon nanotube (CNT) in which boron and nitrogen atoms arranged in a hexagonal network. Owing to the unique atomic structure, BNNT has numerous excellent intrinsic properties such as superior mechanical strength , high thermal conductivity, electrically insulating behavior, piezoelectric property, neutron shielding capability, and oxidation resistance. Since BNNT was first synthesized in 1995, developing efficient BNNT production route has been a significant issue due to low yield and poor quality in comparison with CNT, thus limiting its practical uses. However, many great successes in BNNT synthesis have been achieved in recent years, enabling access to this material and paving the way for the development of promising applications. In this article, we discussed current progress in the production of boron nitride nanotube, focusing on the most common and effective methods that have been well established so far. In addition, we presented various applications of BNNT including polymer composite reinforcement, thermal management packages, piezo actuators, and neutron shielding nanomaterial.

## Introduction

Outstanding and exceptional physical properties of a material can emerge when its size reduces to the nanoscale. Developing nanostructure possessing unique features has always been the essence of nanoscience and nanotechnology since the beginning. Among various nanostructures in general and all kinds of a one-dimensional network in particular, nanotubes have built a strong reputation as the most widely studied nanomaterial. Take carbon nanotube as an example, theoretical and experimental research in every aspect of tubular nanostructure have been flourishing ever since the first discovery of CNT in the early 1990s [1]. CNTs offer numerous fascinating applications in electronic [2], sensing [3], composite [4], and many more are being progressed. The development of new class of nanotube beside the famous carbon allotrope has become an attractive topic in recent years.

**Fig. 1:**
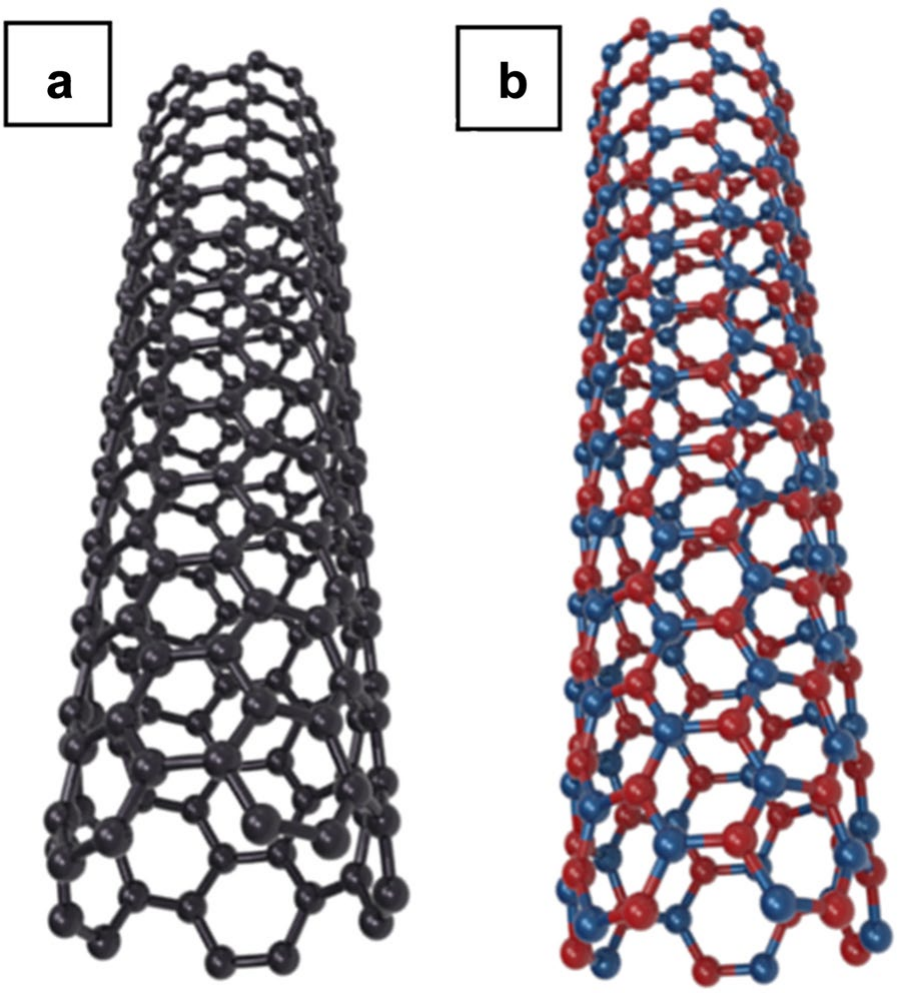
Structural model of **a** CNT and **b** BNNT. The alternating B and N atoms are shown in blue and red, respectively, on the BNNT model

Boron nitride nanotubes (BNNTs) have a similar tubular structure as carbon nanotubes in which carbon atoms are replaced entirely by boron and nitrogen atoms, arranging in a hexagonal lattice (Fig. 1). Not surprisingly, because of this similarity, both BNNT and CNT share some identical intrinsic characteristics, such as excellent mechanical properties, high thermal conductivity [5]. Their Young’s modulus was experimentally measured at a TPa level [6–9]. Although having smaller Young’s modulus and yield strength than those for CNTs, BNNTs were predicted to be thermo-mechanically stable at high temperature [7, 10]. Also, compared to CNTs, BNNTs have similar thermal conductivity [11, 12] and oxidation resistance (up to 900 °C) [13]. On the contrary, while CNTs are semimetallic and semiconducting material [14], BNNTs are an excellent insulator [15] with a wide bandgap (5–6 eV) [16, 17]. The bandgap of BNNT is not dependent on the diameter, and chirality-geometrical information of nanotubes [16], but particularly vulnerable to doping [18–21], and functionalization [22, 23]. The contradiction in electronic property is knowingly attributed to the polarity in B–N bond caused by the difference in electronegativity of boron (2.04), and nitrogen (3.04) [24, 25].

Extraordinary and yet distinct characteristics of BNNT have triggered great interest in fundamental studies on properties and applications of this new exotic material. However, unlike research in CNTs which has been well established over a decade, the study on of BNNT is still immature and far less developed than the carbon counterpart. The reason for this situation lies in the synthesis of BNNT which still remains a significant challenge since BNNT was first discovered in 1995 [26]. Whereas the production of high-quality CNT can be easily done in a laboratory with simple equipment, BNNT synthesis require specially designed apparatus in extreme conditions. The lack of efficient synthesis route combining with the high price of readily commercial products could seriously hinder the study of BNNT in the long term. Many remarkable successes in BNNT synthesis have been recently achieved utilizing newly developed and novel techniques. Large quantity and high quality of BNNT are now becoming accessible, and in turn this will gradually foster BNNT research field. In this review, we will present BNNT synthesis methods that are currently widely used, and applications of BNNTs in various area.

## BNNT synthesis methods

BNNT has been synthesized mainly by methods that have been well documented earlier for CNT fabrication including arc discharge [27], chemical vapor deposition (CVD) [28], laser ablation [29], etc. The essential factor rendering efficient BNNT synthesis process is the rate of conversion from boron and nitrogen sources into BN radicals. Each fabrication technique was scientifically developed in different and distinct strategies, involving specific precursors, conditions, and equipment to promote the growth of BNNT. In this chapter, we will present an overview of various synthesis methods and underline notable features.

### Arc discharge method

**Fig. 2:**
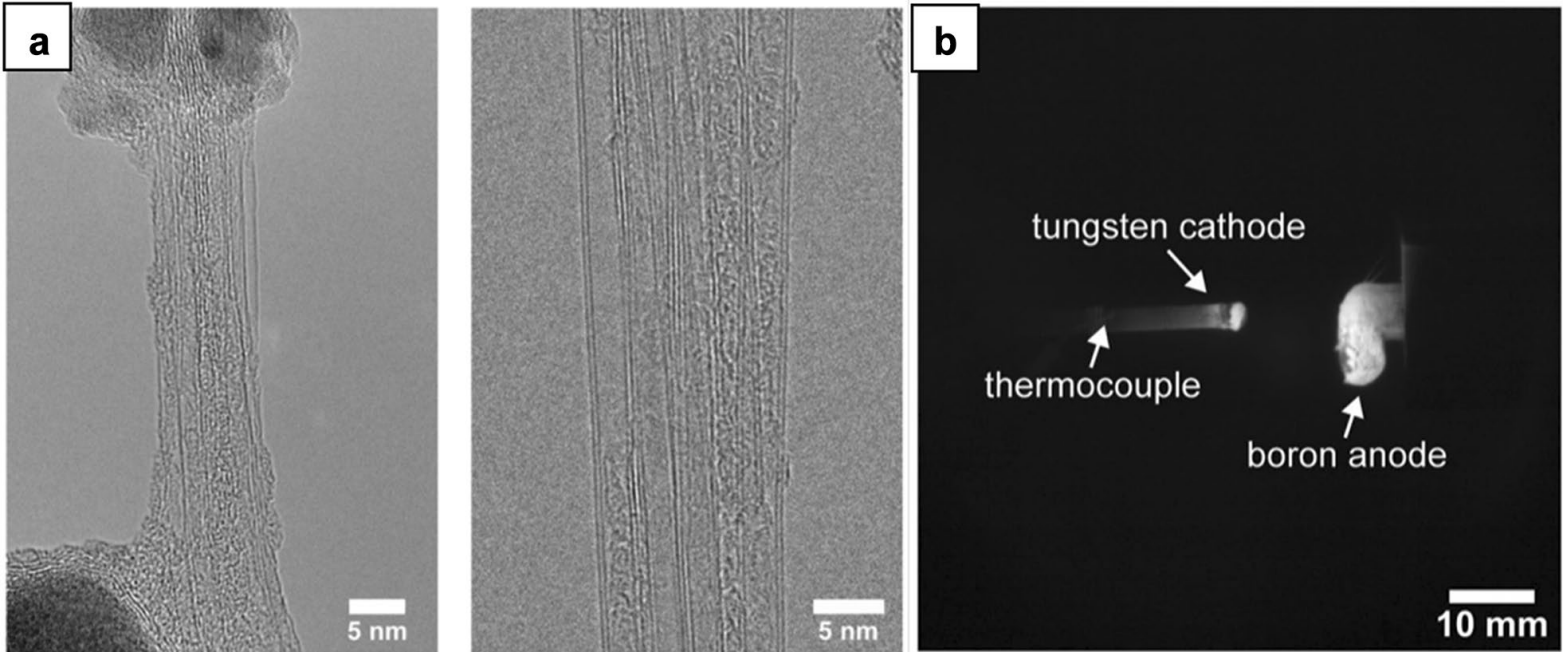
**a** Single- and double-walled BNNT synthesized by arc discharge method. **b** IR image of the cathode and anode electrodes in the chamber. Reproduced with permission from [33]. Copyright 2017, Springer Nature

BNNT was experimentally found in arc discharge for the first time by Chopra et al. in 1995 [26]. A BN rod as a precursor for BNNT synthesis was inserted into a hollow tungsten anode electrode. Arc plasma generated between the cathode and anode quickly vaporized the anode part. Synthesized BNNTs had an inner diameter of 1–3 nm, a length of 200 nm, and one end wrapping a dense particle, probably tungsten or tungsten compound with boron and nitrogen. Because of the insulating property of BN rod that can hamper discharge process, it was later replaced by highly conductive boron-containing chemical compounds (YB_6_, HfB_2_) [30, 31] or a mixture of boron and nickel, cobalt [32] and N_2_ was used as surrounding gas. By doing so, it not only increased the conductivity of anode but also provided catalytic activity toward BNNT growth. Narita et al. obtained BNNTs by arc-melting YB_6_ powder and confirmed the catalytic influence of yttrium on producing few-walled BNNTs (4–10 layers) from the evidence of YB_2_ particles existed at the end of nanotubes [30]. Loiseau et al. used a high-purity, hot pressed HfB_2_ electrode to synthesize BNNT and observed a large number of a nanotube with reduced layer number having flat ends [31]. Cumings et al. obtained abundant double-walled BNNTs in arc discharge when incorporating a small amount of nickel and cobalt as primary catalysts into boron-rich conducting electrode [32]. Regardless of anode materials, the choice of cathode material is flexible as pointed out in a study reported by Yeh et al. [33]. The author found that the quality of BNNTs was similar when arcing tungsten or boron-rich cathode with the anode (Fig. 2a). However, using tungsten or any refractory metal with high melting temperature can stabilize arc plasma (Fig. 2b). The main disadvantage of arc discharge process is that it is hard to produce BNNT at large scale as the reaction zone at the arc core is confined in a small volume.

### Ball-milling method

Ball milling is a promising technique to synthesize BNNT at industrial scale with low cost. In principle, direct reaction between boron and nitrogen in ambient conditions can be stimulated by introducing defective or amorphous structure in boron starting powders. This transformation is easily done by applying sufficient amount of mechanical energy that is controlled by several parameters such as milling time, and intensity (round per minutes). Therefore, the quantity of BNNT can be immensely produced in a typical run. This process is dependent on the milling time that could be extended to hundreds of hours, and the subsequent annealing of treated boron powder has an essential role in the formation of BNNT.

**Fig. 3:**
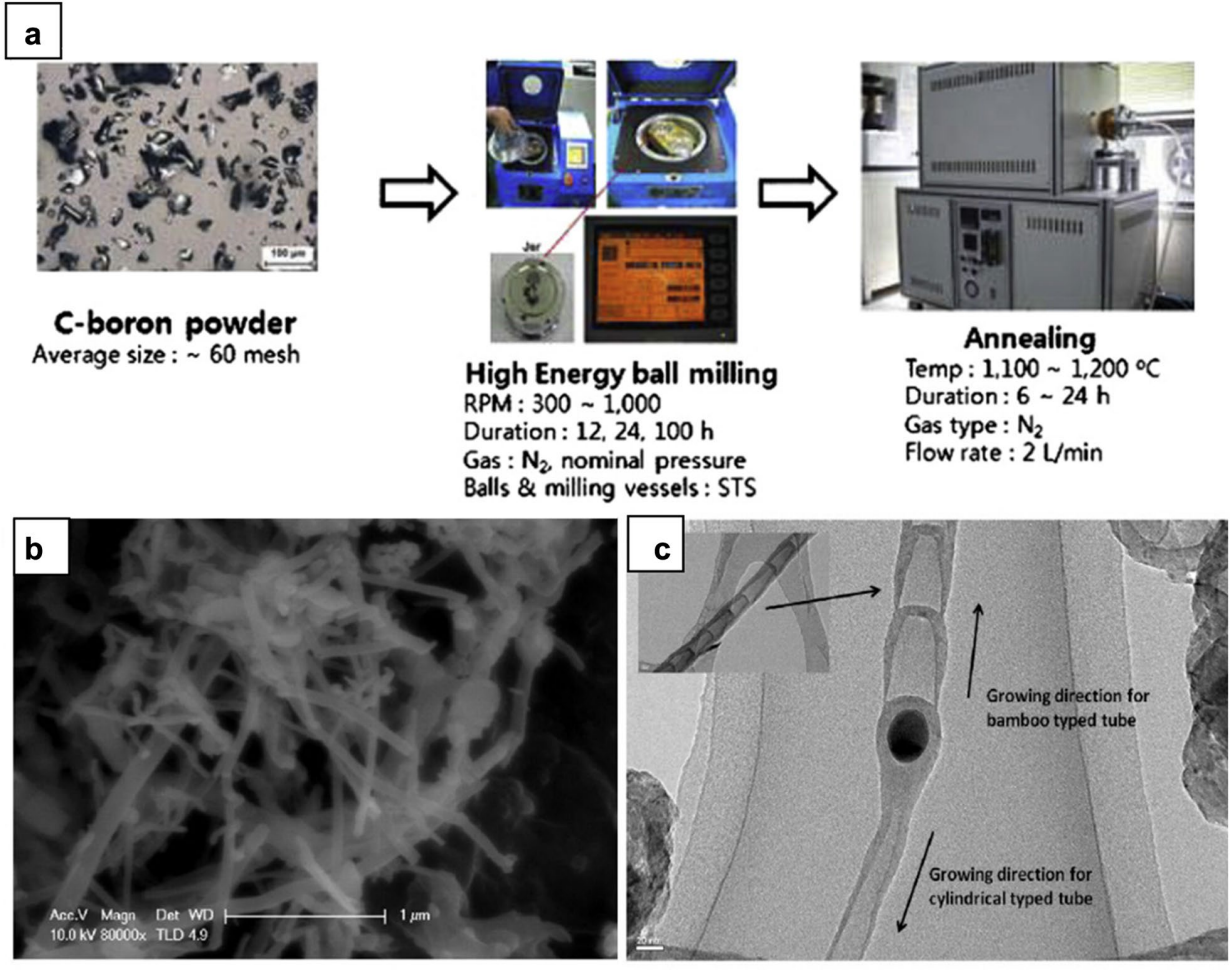
**a** Experimental procedure of ball-millng method. **b** SEM and **c** TEM image of BNNTs produced by the ball-milling method. Cylindrical- and bamboo-like shape of BNNTs is shown in TEM image. Reproduced with permission from [36], copyright 2011 Acta Materialia Inc. Published by Elsevier Ltd. All rights reserved

In a study reported by Chen et al., boron powder was ball milled for 150 h in NH_3_ gas, followed by isothermal annealing at 1000–1200 °C in N_2_ atmosphere [34]. The authors suggested in later article that long milling time would be the crucial factor to achieve a high yield of BNNT as it promoted the nitration process between boron and NH_3_, leading to the formation of increasing nucleation structures that facilitate the formation of BNNT [35]. The optimal annealing temperature proposed in these studies was 1200 °C to ensure the highest conversion rate from nitration products to BN tubular fibers. In addition, milling and heating conditions critically governed the number and size of nanotubes [34]. For instance, increasing milling time and intensity could considerably improve the quantity of BNNTs. Meanwhile, the annealing temperature of 1000 °C favored the formation of thin nanotubes (25–50 nm). During the process, iron particles from milling ball and container dispersed into boron powder can act as an efficient catalyst to accelerate nitriding reaction. Kim et al. observed the growth of BNNT from amorphous boron coated on the surface of the catalytic iron particle originated from stainless steel vessel and claimed that the type of as-grown BNNT was determined by the initial shape of BN clusters on Fe seed particles [36]. In his experiment, crystalline boron powder underwent the transformation into amorphous structure covered the surface Fe particles where it then initiated the growth of BNNTs in annealing phase. No nitriding reaction occurred in milling process since it was conducted in nitrogen gas. Amorphous nanoshells on the surface of spherical iron particles were the initial state of bamboo-like nanotubes, while opened nano cylindrical BN clusters were responsible for the growth of cylindrical nanotubes (Fig. 3). BNNTs with a diameter of few nanometers and unique cylindrical structure were produced when ammonia was employed in annealing process instead of nitrogen [37]. It should be noted that the purity of the final product is relatively low as it commonly comprises amorphous boron and boron nitride flakes along with BNNT.

### Chemical vapor deposition (CVD) method

Chemical vapor deposition (CVD) is one of the most popular ways that have been widely used to produce carbon nanomaterials, and it was lately adopted to synthesize boron nitride materials. In comparison to other approaches, this technique offers better controllability of growth parameters regarding growth mechanism, experimental setup, precursors, catalysts, and temperature, to ensure the high quality of nanomaterials [38]. It is not surprising that CVD process for BNNT growth is relatively similar and reminiscent of CNT synthesis, the differences lie primarily in the types of starting materials, growth conditions. Liquid or solid boron and boron nitride sources are commonly used to grow BNNT instead of toxic and combustible gaseous boron precursors, along with nitrogen gases, such as N_2_ or NH_3_. Also, similar to CNT synthesis, transition metal catalysts are particularly efficient at producing BNNT with few layers and small diameter.

**Fig. 4:**
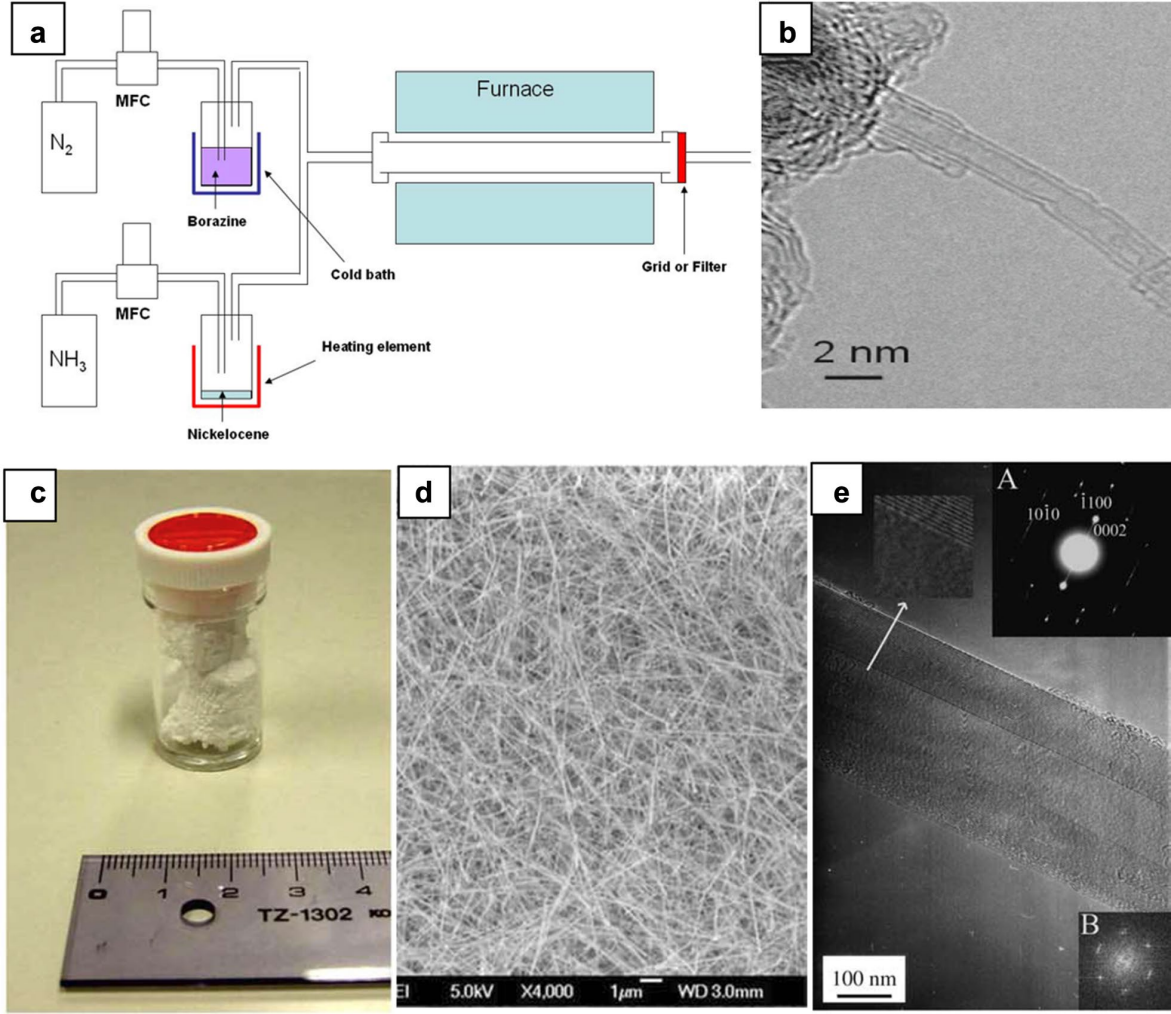
**a** Schematic of the catalytic chemical vapor deposition (CCVD) and **b** TEM image of the double-walled BNNT produced by CCVD method. **c** 200 mg of BNNTs synthesized by boron oxide chemical vapor deposition (BOCVD) at 1500 °C over 1 h. Images of **d** SEM and **e** TEM of as-grown BNNTs by BOCVD, inset A and B in **e** are corresponding SAED and FFT patterns. (**a**, **b** Reprinted with permission from [40], copyright 2008 American Chemical Society. **c**–**e** Reproduced with permission from [44], copyright 2005 Elsevier B.V. All rights reserved)

The first attempt to synthesize BNNT by CVD process was carried out by Lourie et al. by using borazine generated from the chemical reaction between (NH_4_)_2_SO_4_ and NaBH_4_, as boron nitride source, combining with metal catalysts such as Co, Ni, NiB or Ni_2_N at temperature of 1000–1100 °C [39]. Attained BNNTs mostly had a multi-walled structure with tube length up to ~ 5 µm and irregular caps (bulbous, club-like, flag-like). In another study reported by Kim et al. purified borazine source were jointly used with floating catalyst nickelocene to prevent the formation of unnecessary BN species or probable chemical contamination (Fig. 4a) [40]. Detailed experimental setup was reported in which two gas streams: (1) NH_3_ carrying nickelocene as the catalyst, (2) borazine containing N_2_ gas, flew into the hot zone of the furnace. The growth of BNNT was controlled by flow ratio of ammonia to nitrogen (100:3 sccm) at 1200 °C. As grown BNNTs acquired from this approach were primarily dominated by double-walled nanotubes with 2 nm diameter and several hundreds of nanometer in length (Fig. 4b).

Although almost all CVD processes are involved in the use of the catalyst, it is possible that non-catalytic approach can be applied to grow BNNT. Ma et al. attempted to synthesize BNNT using B–N–O precursor generated from melamine diborate (C_3_N_6_H_6_·2H_3_BO_3_) [41]. The powder precursor was then rapidly heated and kept in the N_2_ atmosphere for 2 h. Multi-walled BNNTs formed after the reaction had concentric layers with an inner and outer diameter of 5.2 and 13.1 nm, respectively. The tip of nanotubes was bulbous-like shape encasing B–N–O clusters. In another similar work, BNNT grew directly on α-Al_2_O_3_ Å µm-range particles, showing 2–6 concentric layers with slightly larger diameter and partially filled by amorphous B–N–O or boron carbide crystalline [42].

In contrast to the hundred-gram scale of CNT that can be easily produced, the yield of BNNT obtained in CVD process remains significantly low, typically a couple of 100 mg. Therefore, in pursuance of mass producing BNNT, Tang et al. have developed the so-called boron oxide CVD (BOCVD) in which boron and MgO powder were used as reactants to generate B_2_O_2_. BNNTs were subsequently formed via the reaction between B_2_O_2_ and NH_3_ gas at a temperature ranging from 1000 to 1700 °C [43]. The diameter of BNNT formed in this way varied in a wide range from several nanometers to 70 nm, influencing on the constitution of defects. All nanotubes possessed parallel fringe pattern, indicating multi-walled structure with a length up to 10 µm. Gram scale of BNNT having comparable quality can be achieved with the utilization of FeO in the mixture of B and MgO (Fig. 4c–e) [44]. It was later found that Li_2_O could produce better effects than MgO did on the large-scale production of BNNTs with the reduced diameter of sub-10 nm due to its superior deoxidation capability and pronounced promotion effect on the crystallization of graphite-like BN [45].

### Laser ablation method

Fabrication of BNNT in laser ablation method has the advantage of producing high-quality nanotubes with a small number of the wall, high aspect ratio, and crystallinity. In this method, a target made of boron or boron nitride undergoes a phase transformation from solid to liquid at a high temperature that exceeds boron melting point (2000 °C) due to laser heating. Thus, direct reaction between surrounding nitrogen atmosphere and boron target can be enhanced efficiently, resulting in the efficient growth of BNNTs. Golberg et al. succeeded in synthesizing BNNT using laser ablation method for the first time [46]. Multi-walled BNNTs were synthesized by laser heating cubic and hexagonal BN targets until 5000 K in a diamond anvil cell in extremely high-pressure (5–15 GPa) of nitrogen gas.

Yu et al. synthesized BNNT by irradiating laser to target prepared by mixing Ni–Co catalyst particles with h-BN powder similar to CNT synthesis [47]. As grown BNNTs had 1–3 layers, and a diameter ranging from 1.5 to 8 nm. Also, through TEM analysis, two types of nanotubes were identified in which BNNT grown with and without catalyst particles nested in tube ends. Zhou et al. confirmed the role of Ni–Co in the following study [48]. In the absence of Ni–Co catalyst, BNNT showed a multi-walled structure with a diameter of 1.5–6 nm and impurities irregularly distributed on the surface of the nanotubes. Whereas in opposite case, most of BNNTs were single walled and impurity free. They explained that the Ni–Co catalyst plays a role in synthesizing high-quality single-walled BNNT.

Lee et al. at Office National d’Etudes et de Recherches Aérospatiales (ONERA) and Centre national de la recherche scientifique (CNRS) in France have developed a method for synthesizing BNNTs using catalyst-free h-BN as a source in by laser ablation [49]. Gram scale of BNNTs (0.6 g/h) was synthesized by heating the target at 3400 K using the continuous CO_2_ laser in atmospheric nitrogen pressure. The obtained sample consisted of single-walled BNNTs tied into bundles with a length of about 100 nm, besides double and multi-walled tubes. Two ends of nanotube were either encapsulated by boron nanoparticles or flat-closed. This evidence suggests that the formation of BNNT can follow root growth mechanism. Besides, the growth of single-walled BNNTs without catalyst was also confirmed. Next, in another study conducted by Arenal et al. in ONERA-CNRS, BNNTs were produced by continuously heating pressed a mixture of h-BN powder and boron oxide binder as a target in nitrogen partial pressure conditions [50]. The amount of single-walled BNNTs, in this case, was account for roughly 80% in final products, and tube length was several 100 nm. From the analysis of boron nanoparticle enclosed by h-BN layer, it was found that oxygen impurities could restrain the formation of BNNT. In situ diagnostic methods developed by Cau et al. using the UV-laser induced fluorescence (LIF) and UV–Rayleigh scattering (RS) indicate the existence of BN and BO species in the plume above heated h-BN target [51]. The results supported the root growth mechanism and confirm the inhibition of BNNT growth due to oxygen impurities in previous studies. Therefore, to increase the yield of BNNT, pure boron and nitrogen sources are highly required.

**Fig. 5:**
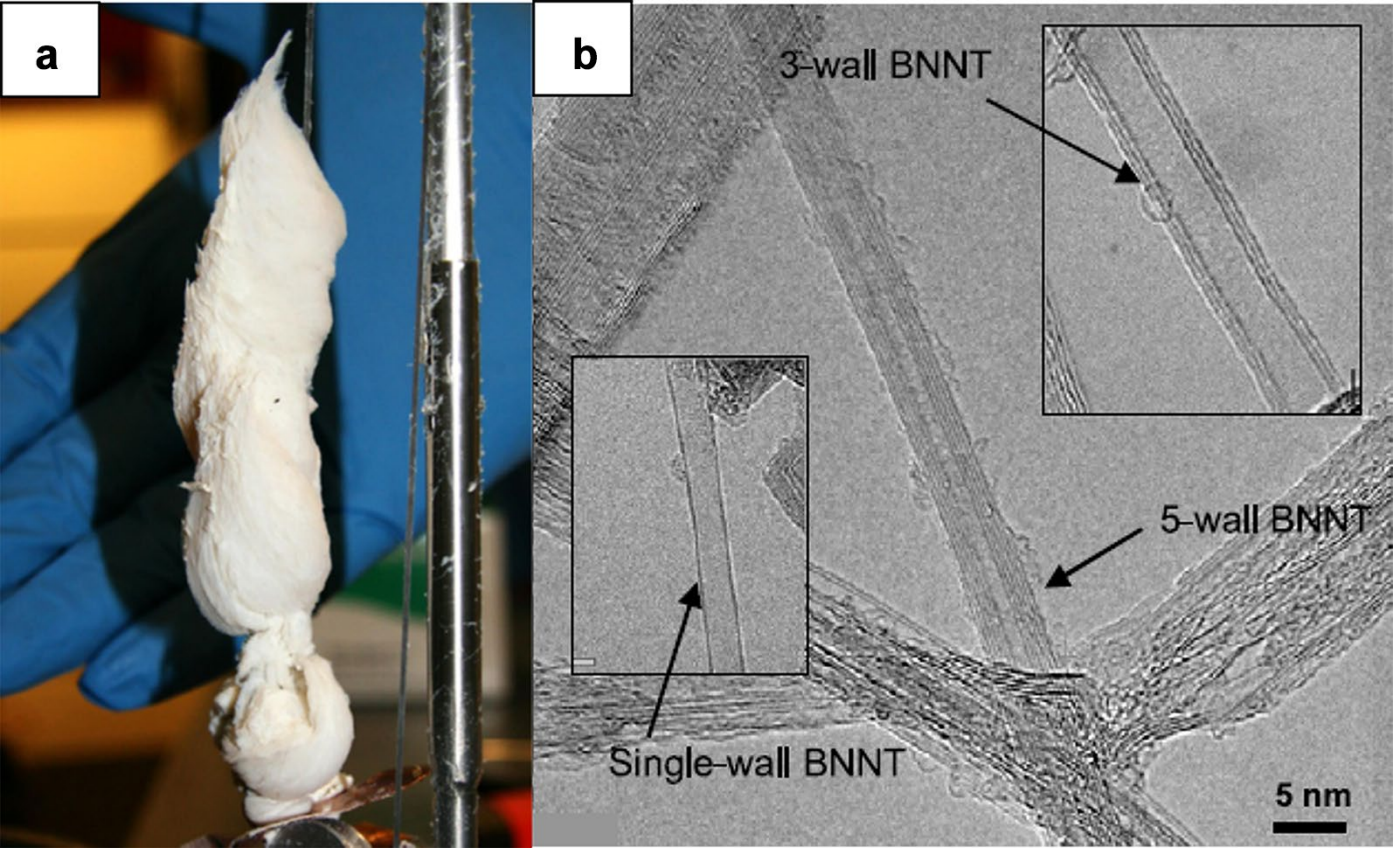
**a** 200 mg of BNNTs synthesized by the laser ablation method in a one production process. **b** TEM image of one-, three- and five-walled BNNTs. (Reprinted with permission from [52], Copyright 2009 Institute of Physics Publishing)

NASA Langley Research Center synthesized BNNTs under high temperature/pressure conditions using boron metal fibers and nitrogen gas [52]. The reaction chamber was filled with nitrogen gas at 14 bar, and the boron metal fiber target was continuously heated by the CO_2_ laser (~ 4000 K). This method was able to synthesize gram scale of high purity BNNTs with a production rate of ~ 0.2 g/h, small diameter (< 5 nm) and 2–5 layers (Fig. 5). A simulation study on hot plumes generated above boron fiber target during laser ablation process demonstrated that the high yield of BNNTs was achieved by the supersaturated BN vapor and recirculation region at the base of the plume at high temperature and pressure conditions [53].

### Thermal plasma jet method

Though BNNTs produced by laser ablation technique possessed very high structural quality, the production rate is still limited, under a milligram per hour, due to the confinement of growth area in a small volume. Thermal plasma methods seem to be an alternative solution to this issue, since it is capable of applying high thermal energy over a wider volume, extending growth area of BNNTs up to hundreds square centimeters. Plasma jet, one of the thermal plasma-based methods, consists of two concentric electrodes anode and cathode. The plasma generated when a gas mixture (Ar, N_2_, H_2_) flows in between two electrodes pass through a nozzle, forming a broad region of arc plasma jet [54].

Shimizu et al. prepared multi-walled BNNTs by heating a sintered BN target in a plasma jet (DC power of 8 kW, Ar–H_2_ mixed gas) at 100 Torr (~ 4000 K) [55]. The results indicated a strong correlation of high-temperature conditions with mass production of BNNT in this method. Through the TEM analysis, some initial evidence on the growth direction and mechanism of BNNT from phase boundaries existing at the opposite ends of nanotube was given. Lee et al. synthesized BNNTs by continuously injecting a powder mixture of h-BN and Ni/Y catalyst particles into a plasma jet (DC power of 14 kW, Ar–N_2_ mixed gas) at atmospheric pressure (5000–20000 K) [56]. Though most of the BNNTs were identified as multi-walled with diameters ranging from 3 to 10 nm, single and double walled BNNTs were also observed.

**Fig. 6:**
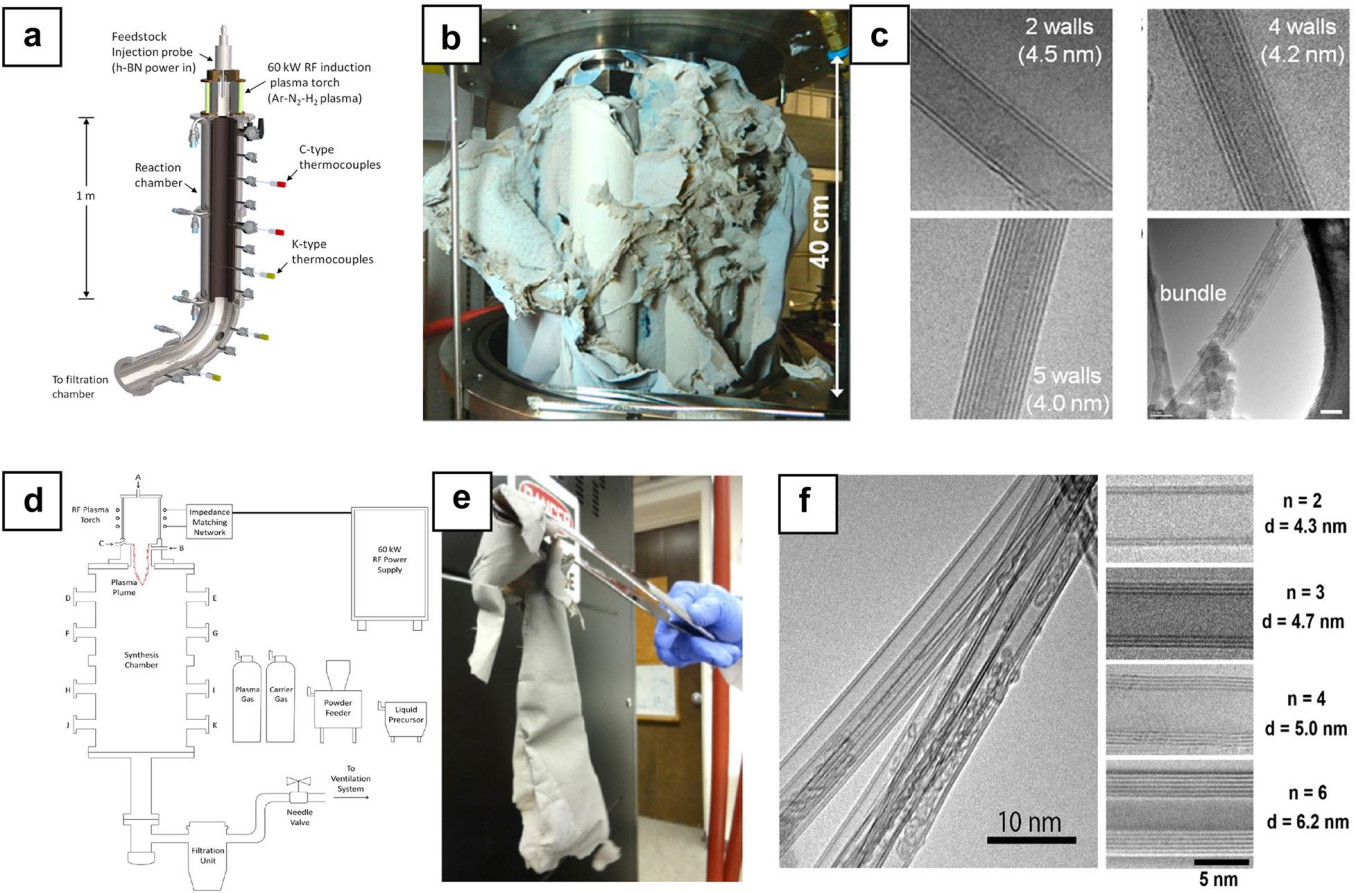
Scalable synthesis of BNNTs by plasma jet process. **a** Schematic of induction plasma processing system, **b** 192 g of BNNT materials produced in single process (20 g/h) and **c** TEM analysis on as-grown BNNT materials. **d** Schematic of extended pressure inductively coupled (EPIC) thermal plasma system, **e** BNNT materials produced at the rate of 35 g/h, and **f** TEM images of as-grown BNNTs by EPIC synthesis. (**a**–**c** Reproduced from [57], copyright 2014 American Chemical Society. **d**–**f** Reprinted with permission from [59]. Copyright 2014 American Chemical Society)

Recently, a group at the National Research Council Canada (NRC) has successfully achieved a very high production rate of BNNT by using RF induction plasma jet process [57]. This method can induce a large area comprising slow plasma gas, thus increasing the residence time of precursors in a highly energetic environment where BNNT growth takes place. Plasma was generated by Ar gas and stabilized by a gas mixture (Ar/N_2_/H_2_). The h-BN powder was subsequently infused into plasma torch by Ar gas (Fig. 6a). The production rate was significantly high, approximate 20 g/h (Fig. 6b), and the few-walled and highly crystalline BNNTs obtained in this process had an average diameter of 5 nm (Fig. 6c). Additionally, this study also suggested that hydrogen gas can act as a catalyst for the efficient growth of BNNT. With the addition of hydrogen, the B–N–H was formed and promoted the formation of BNNT by efficiently reacting with the boron nano-droplet rather than nitrogen molecules. Hydrogen effect was investigated through additional simulations and in situ optical emission spectroscopy (OES) study [58]. Zettle et al. obtained double-walled BNNTs at very high production rate (35 g/h) while developing the extended pressure inductively-coupled plasma system (EPIC) method in high-pressure conditions based on induction plasma method (Fig. 6d, e) [59]. Using specially designed EPIC system capable of generating plasma at high pressure, synthesis process was carried out by injecting boron or h-BN powder into a plasma jet of 7 MHz RF power between 40 and 50 kW at maximum nitrogen pressure of 10 atm. 70% of synthesized BNNTs are double-walled nanotube with a diameter of 4 nm (Fig. 6f). BN nano-cocoon and BN nano-ribbon byproducts were also found. From this study, it is worth to mention that hydrogen gas is not necessary for the promotion of B–N_2_ reaction which is particularly vital to achieving a high yield of BNNT.

### BNNT applications

Based on various unique properties, BNNTs have great potential for practical uses in many areas. Despite limitations on fabrication technique, there is an increasing number of fundamental studies, suggesting and developing BNNT applications. Significant successes have been achieved, and in most case, BNNT is conclusively proved to be a promising candidate for mechanical reinforcement in the nanocomposite and advanced functional materials.

**Fig. 7:**
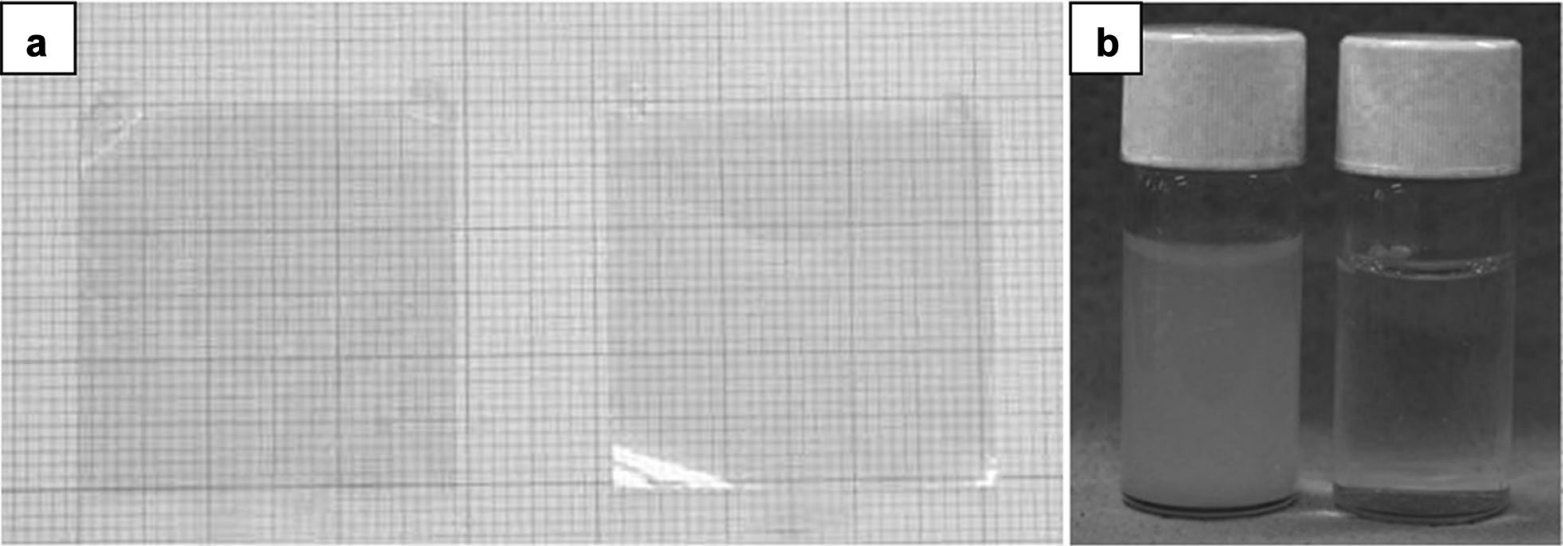
BNNT application for polymer composite reinforcement. **a** PS film (left), and a BNNT/PS composite film with a 1 wt% BNNT fraction (right). **b** An aqueous solution of hydroxylated BNNTs (left), and pristine BNNTs in water (right). (**a** Reproduced with permission from [60], copyright Materials Research Society 2006. **b** Reproduced with permission from [61], copyright 2009, John Wiley and Sons)

BNNTs exhibit high mechanical strength, and elastic modulus comparable to those of CNTs, but thermomechanical stability is superior to its carbon counterpart at high temperature. To exploit these properties, BNNT/polystyrene composite films had been made and mechanical tests on the fabricated membrane were conducted [60]. This study showed that the composite displayed perfect transparency with the elastic modulus increased by 21% when 1 wt% of BNNT was mixed (Fig. 7a). Compared to the blank polymer, the composite has better oxidation stability and slightly lowered glass transition temperature. On the other hand, to reinforce the composite, BNNTs should be well dispersed and strongly bonded with the polymer matrix. Therefore, in most cases, modified or functionalized BNNT is a better choice than primary material due to the higher affinity with polymers, resulting in enhanced interfacial interactions between the two. In another report, the mechanical properties of polymeric composites of pristine BNNTs and hydroxylated BNNTs were measured [61]. The intrinsic properties of BNNTs were changed in which, in contrast to the hydrophobicity of pristine nanotube, BNNTs turn to water-soluble after being functionalized (Fig. 7b). As a result, the modulus of elasticity increased by 20% when untreated BNNT was added to both polymers but further increased by more than 30% after using hydroxylated BNNT.

**Fig. 8:**
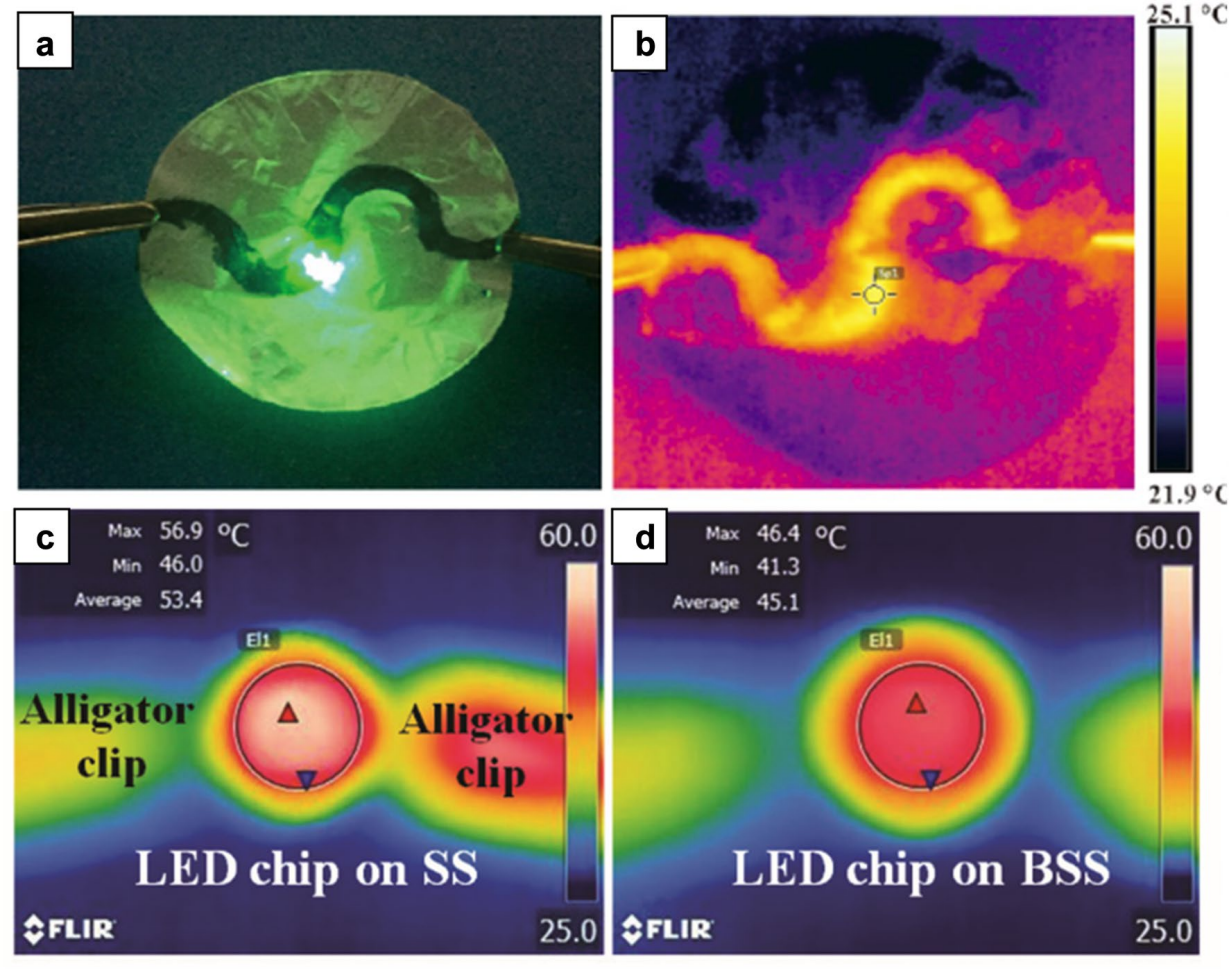
BNNT application for thermal management packages. **a** Optical and **b** thermal photographs of working electronic devices using CNF/BNNT nanocomposites as a printed circuit board. Thermal photographs of the fabricated GaN LED chips, **c** without and **d** with the BNNTs taken after 1 h operation under a continuous injection current of 100 mA. (**a**, **b** Reprinted with permission from [63]. Copyright 2017 American Chemical Society. **c**, **d** Reproduced from [64] with permission from the Royal Society of Chemistry)

In virtue of high thermal conductivity exclusively governed by phonons, BNNTs can be integrated with various polymers to create a new class of thermal conductive BNNT-polymer composite for packaging material or heat-releasing substrate applications. Zhi et al. successfully achieved more than 20-fold thermal conductivity improvement in BNNT-PMMA polymeric composite, while holding excellent electrical insulating properties [15]. Jakubinek et al. performed the first direct measurement of thermal conductivity for pure BNNT-sheets [62]. A value of 1.5 Wm^−1^ K^−1^ was recorded for filtration-produced BNNT buckypaper, and it was 0.75 Wm^−1^ K^−1^ for low-density as-synthesized sheets. The thermal conductivity of buckypaper was solidly affected by tube–tube contact resistance, bundling, and tube misalignment. They also found that thermal conductivity of epoxy-impregnated BNNT buckypaper composite (30 wt% BNNT) was 10 times higher than the epoxy. Zeng et al. used cellulosic nanofibers (CNF) and BNNT as constituents of flexible thermal conductive nanocomposites (Fig. 8a, b) [63]. The strong interaction of nanofiber with BNNTs was established by non-covalent functionalization without damaging nanotubes. The thermal conductivity of composite was remarkably high (21.39 W/mK at 25 wt% BNNTs), owing to the high intrinsic thermal conductivity of BNNTs and CNFs. Recently, research on the use of BNNT as heat sink layer in electronic devices has been reported. It is known that heat generated by electronic devices can increase the junction temperature, reducing the working efficiency and shortening the lifespan of devices. Therefore, applying nanomaterials with excellent thermal conductivity in the fabrication of high power electronic devices is a viable solution to deal with heat generation. Seo et al. incorporated BNNT within GaN LEDs to examine thermal dissipation performance [64]. The LEDs were made by growing high-quality GaN epilayer on BNNT-deposited sapphire. Compared to conventional LEDs without BNNTs, the fabricated GaN LEDs had notably lower surface temperature, thanks to the heat spreading BNNT layer (Fig. 8c, d).

**Fig. 9:**
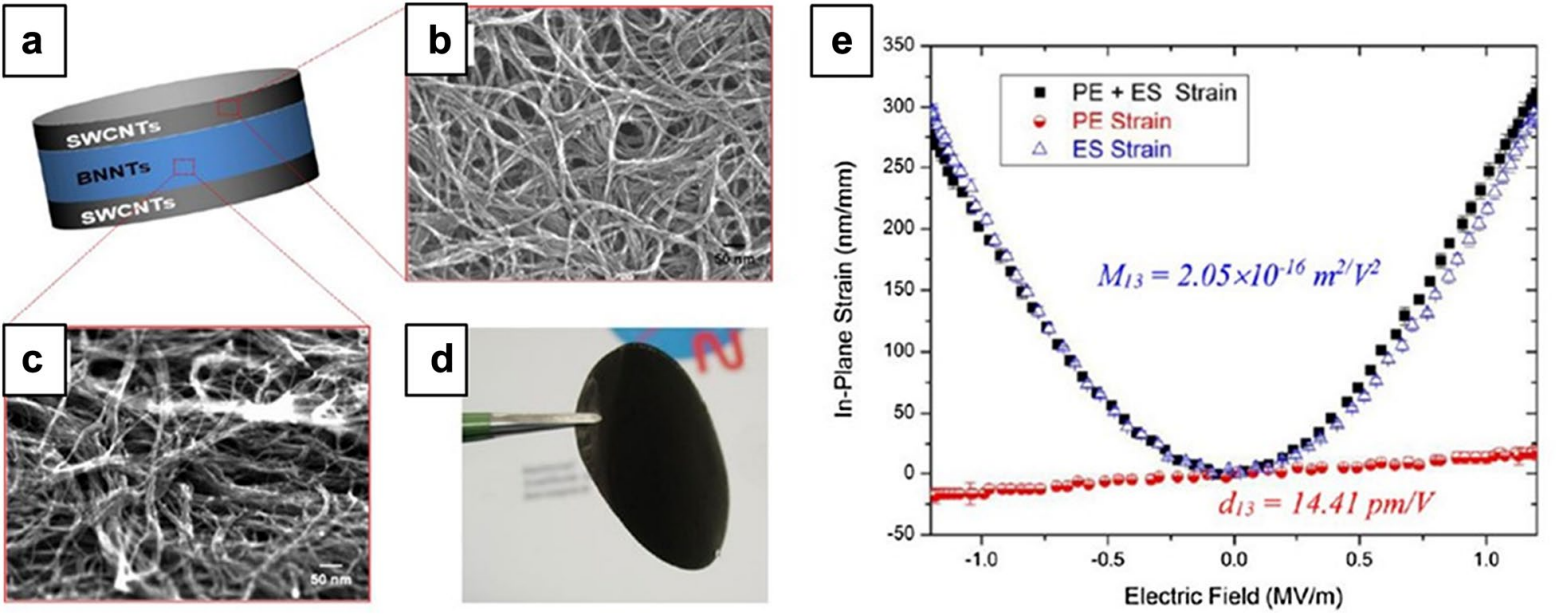
BNNT application for piezo actuators. **a** Schematic structure of the C-/BN-nanotubes piezo actuator. **b** SEM images of the top and bottom SWCNT electrodes. **c** SEM image of the BNNT active layer. **d** Photograph of free-standing of C-/BN-nanotubes piezo actuator consisting of BNNT active layer and SWCNT electrodes. **e** Electroactive performance of the C-/BN-nanotubes piezo actuator. (Reprinted with permission from [66]. Copyright 2015 American Chemical Society)

The piezoelectric properties of BNNT have been identified in several studies, indicating the broad bandgap nature is tunable under deformation. In a theoretical work done by Nakhmason et al., it was suggested that BNNTs are excellent nonpolar piezoelectrics that exhibit substantially higher strain response than polar polymer [65]. Kang et al. fabricated novel multifunctional electroactive nanocomposites using BNNT and polyimide [66]. Under the influence of the external electric field, the piezoelectric coefficient (d_33_) and electrostrictive coefficient (M_33_) of 2 wt% BNNT/polyimide composite were − 0.84 pm/V and − 3.07 × 10^−19^ pm^2^/V^2^, respectively. Both coefficients significantly increased by about 460% when BNNTs were aligned by stretching the composite. Especially, when combining a BNNT buckypaper layer in between two single walled CNT electrode layers in an all-nanotube sandwich-like structural actuator, the piezoelectric coefficient (d_13_) and electrostrictive coefficient (M_13_) were far larger (14.41 pm/V and 2.05 × 10^−16^ pm^2^/V^2^, respectively) (Fig. 9).

**Fig. 10:**
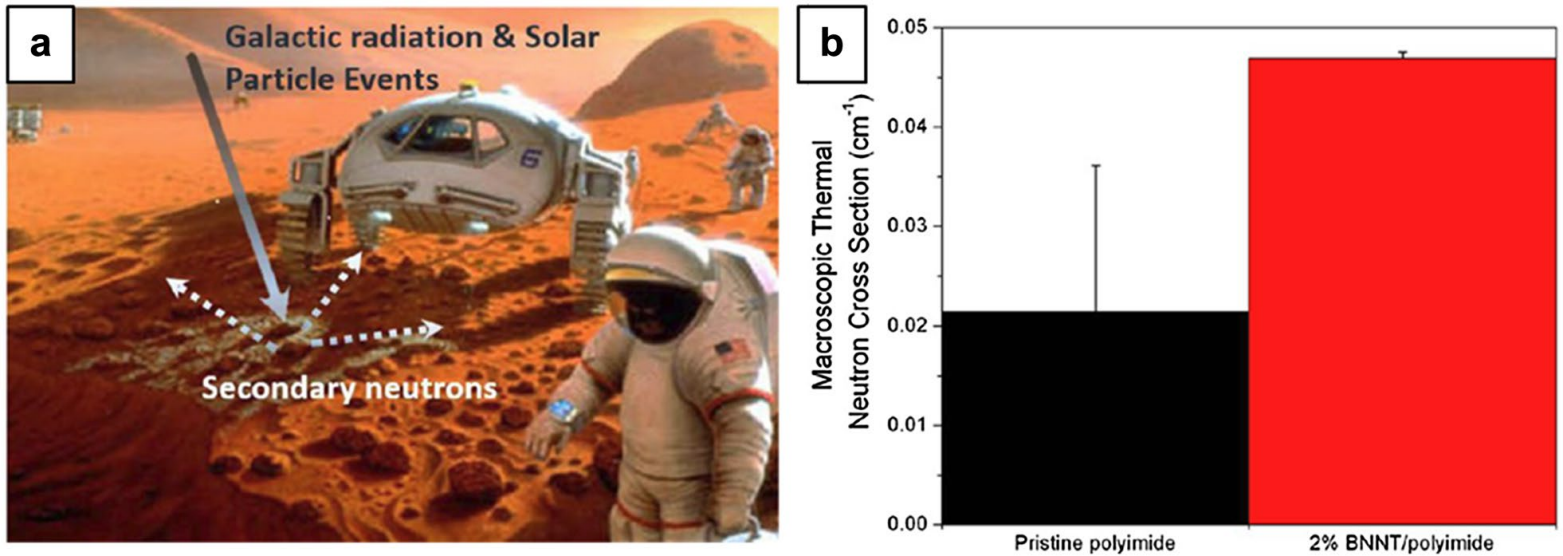
BNNT application for neutron shielding nanomaterial. **a** Conceptual space exploration illustration. **b** Macroscopic thermal neutron cross sections of pristine polyimide and 2 wt% BNNT/polyimide composite. (**a**, **b** Reprinted with permission from [67]. Copyright 2015 American Chemical Society)

In space exploration, neutron absorption has become a significant concern as neutron mostly generated from the interaction of cosmos radiation with matter can cause disastrous malfunction or lethal damage to space equipment and astronauts (Fig. 10a). Owing to high neutron absorption cross-section of 3835 barn, BNNTs compounded of isotope ^10^B is a promising nanomaterial for shielding from neutrons in cosmic environment. Kang’s group at NASA studied BNNT-polymer composite films for neutron shielding [66]. A neutron cross section of polyimide having neutron moderator characteristics is 0.021 cm^−1^. Due to neutron capture capability of boron, the neutron cross-section of 2 wt% BNNT/polyimide films was 0.047 cm^−1^, increasing by 120% in comparison with pristine polyimide (Fig. 10b). Current interest has shifted toward hydrogen-containing nanotube since the hydrogen content in the material can improve the radiation-shielding effectiveness against space radiation including solar particle events (SPEs), galactic cosmic radiation (GCR) [67]. As explained by Thibeault, due to the higher surface area and higher hydrogen binding energy, nanotubes are preferential to store hydrogen rather than 0-dimensional particles and 2-dimensional sheets [68].

At present, the research study on practical uses of BNNT is extensively and continually conducted. For instance, in water purification, BNNTs has been examined in oil-filtering [69], self-cleaning membranes [70], and reusable heat-resistive films [71]. In the field of biological application, due to the nontoxicity property, BNNTs can be used as a biological probe [72], drug carrier [73], or biological channel in biosensor [74]. High stability towards oxidation of BNNTs has enabled its access to field emission technology [75, 76]. In addition, BNNT has also been reported in hydrogen storage [77–80], sensing [81, 82], and optoelectronic [83, 84]. It is highly anticipated that the application of BNNT will continue to extend in the future.

## Summary

Major achievements in the production of boron nitride nanotube in recent times are the favorable outcome of the development of novel approaches including boron oxide-chemical vapor deposition (BOCVD), thermal plasma, and high temperature–pressure laser ablation. The typical strategy sharing among these methods is to stimulate the direct reaction between boron and nitrogen precursors. This can be done systematically by several ways such as creating highly disorder or amorphous structure in starting materials (ball milling), utilizing effective catalysts (floating catalyst CVD), and producing gas-phase (BOCVD) or liquid-phase (HPC laser ablation) reaction between boron and nitrogen gas. Although the quality and quantity of BNNT have been significantly improved, these approaches also exert side effects, for example, undesirable formation of amorphous boron, boron nitride, and h-BN, thus, leading to the degradation of BNNT purity. To tackle this problem, besides developing an efficient purification process, optimizing synthesis method is critically important. However, in doing so, it requires a profound understanding of the growth mechanism of BNNT in every single method. Recently, computer simulation and in situ diagnostic tools to evaluate every critical factor from several research groups have initially provided some insight into growing process of BNNT [51, 53, 58]. On the other hand, the widespread availability of BNNT has triggered great interest in the development of BNNT applications. The effectiveness of BNNT has been promisingly approved in reinforcing polymeric composite, boosting thermal managing capability of electronic devices, and enhancing neutron shielding in aerospace. Although the BNNT-related technology is still in its infancy, it is expected that many more fascinating applications will be developed in years to come.
